# Deconstructing Ataxia with Oculomotor Apraxia: Toward a Gene‐Based Classification and a Reappraisal of the Oculomotor Phenotype

**DOI:** 10.1002/mdc3.70846

**Published:** 2026-10-02

**Authors:** Luiz Eduardo Novis, Flávio Moura Rezende Filho, Anna Maria Gomes, Victor Rebelo Procaci, Orlando Graziani Povoas Barsottini, José Luiz Pedroso

**Affiliations:** ^1^ Universidade Federal de São Paulo Escola Paulista de Medicina, Departamento de Neurologia São Paulo Brazil

**Keywords:** ataxia, ataxia‐telangiectasia, neurogenetics, apraxia, oculomotor apraxia

## Introduction

Ataxia with oculomotor apraxia (AOA) designates a group of autosomal recessive cerebellar ataxias (ARCAs) that share defective DNA strand‐break repair or RNA processing and a clinical core of progressive cerebellar ataxia, peripheral neuropathy, and variably present oculomotor disturbance. The group currently comprises five numbered subtypes (AOA1–5), caused by biallelic variants in *APTX*, *SETX*, *PIK3R5*, *PNKP*, and *XRCC1*, respectively.^1^, ^2^, ^3^, ^4^ Ataxia‐telangiectasia (AT, *ATM*) and ataxia‐telangiectasia‐like disorder (ATLD, *MRE11A* and ATLD‐2, *PCNA*) share the same repair mechanisms and oculomotor phenomenology but have traditionally been classified separately.^5^, ^6^

We contend that the current classification carries three unresolved problems. The first is one of scope: it excludes AT and ATLD despite their mechanistic and phenotypic overlap with the canonical subtypes, while granting formal subtype status to AOA3, which was reported in a single consanguineous family and never replicated.^1^, ^7^ The second is one of emphasis: the disease name foregrounds a single oculomotor sign, whereas the eye‐movement disturbance in these disorders is considerably broader.^8^, ^9^ The third is one of consistency: that sign is absent in a substantial proportion of patients — roughly one‐third to two‐thirds, depending on subtype—which raises the question of whether it should remain a diagnostic criterion.^2^, ^8^, ^10^ This Viewpoint addresses each problem in turn and proposes a frequency‐ordered, gene‐based classification of the spectrum.

## A Frequency‐ and Gene‐Based Classification of the AOA Spectrum

The traditional classification numbers five subtypes (AOA1–5) in the order their causative genes were identified, without a unifying biological or epidemiological rationale.^5^, ^11^ Yet all entities in the spectrum share progressive cerebellar ataxia, cerebellar atrophy, and axonal sensorimotor neuropathy, with alpha‐fetoprotein (AFP) as the principal discriminating serum biomarker.^5^, ^6^ We propose a framework with three elements. It adopts gene‐based nomenclature; it orders entities by their relative prevalence across international ARCA cohorts, yielding an intuitive differential‐diagnostic hierarchy; and it incorporates AT and ATLD as first‐rank members, on the grounds of shared mechanism and phenotype (Table 1).

**TABLE 1:** Proposed Revised Classification of the AOA Spectrum, Ordered by Prevalence

Current Name	Gene	Proposed Gene‐Based Name	SCAR Designation	Molecular Pathway	Selected References
**AT**	*ATM* (11q22–23)	*ATM*‐related ataxia	—	DNA double‐strand break repair (ATM kinase)	Levy & Lang 2018 (Mov Disord)^12^
**AOA2**	*SETX* (9q34)	*SETX*‐related ataxia	SCAN2	RNA:DNA helicase / R‐loop resolution	Anheim et al 2009 (Brain)^3^
**AOA4**	*PNKP* (19q13)	*PNKP*‐related ataxia	—	DNA strand‐break terminus processing	Bras et al 2015 (Am J Hum Genet)^13^
**AOA1**	*APTX* (9p13)	*APTX*‐related ataxia	SCAR1	DNA single‐strand break repair (aprataxin)	Le Ber et al 2003 (Brain)^14^
**ATLD1**	*MRE11A* (11q21)	*MRE11A*‐related ataxia	—	DNA double‐strand break repair (MRN complex)	Bajek et al 2023 (Front Neurol)^15^
**ATLD2**	*PCNA* (20p12)	*PCNA*‐related ataxia	—	DNA replication/repair (sliding clamp)	Magrino et al 2023 (J Biol Chem)^16^
**AOA5**	*XRCC1* (19q13)	*XRCC1*‐related ataxia	SCAR26	DNA single‐strand break / base excision repair	Hoch et al 2017 (Nature)^4^

*Note*: Entities ordered from most to least prevalent, and collectively designated DNA damage response (DDR) ataxias. AOA3 (PIK3R5) is not included as it is considered a candidate association pending replication. SCAR: spinocerebellar ataxia, recessive (OMIM designation).

Epidemiological data supports this reordering. In the global analysis by Rossi et al. and the Brazilian AOA cohort of da Costa et al., *SETX*‐related ataxia (AOA2) is the most common classic subtype, followed by *PNKP*‐related ataxia (AOA4)—particularly prevalent in Portugal and Brazil—whereas *APTX*‐related ataxia (AOA1) is comparatively infrequent.^1^, ^16^ Ataxia‐telangiectasia (*ATM*‐related ataxia) is among the most prevalent DNA‐repair ataxias worldwide and is consistently represented in AOA‐focused cohorts, yet has been excluded from the AOA rubric for historical rather than biological reasons.^1^, ^5^, ^8^, ^12^ ATLD (*MRE11A*‐related ataxia) and the ultra‐rare *XRCC1*‐ (AOA5) and *PCNA*‐related (ATLD2) forms complete the spectrum.^14^, ^17^, ^18^ Nomenclature and clinical details for each entity are given in Table 1 and S1.

Grouping the spectrum this way invites a collective name, and we would suggest one. Every entity here is a disorder of the DNA damage response (DDR): ATM and the MRN complex sense and transduce double‐strand breaks; aprataxin, PNKP and XRCC1 process single‐strand break termini; PCNA coordinates replication‐associated repair; and senataxin resolves R‐loops that would otherwise threaten genome stability during transcription.^19^, ^20^ *DNA damage response ataxias* (DDR ataxias), with entities named by gene, is mechanistically accurate, covers both single‐ and double‐strand break pathways, and privileges no single clinical sign. AOA can of course be retained as familiar shorthand at the bedside.

AOA3 stands apart. It was identified in a single consanguineous family with a homozygous *PIK3R5* variant and a phenotype resembling AOA2; biological plausibility is supported by cerebellar *PIK3R5* expression, but the gene acts in phosphoinositide signaling rather than DNA repair, and no further cases have appeared in any cohort.^1^, ^7^ Consistent with standard practice in human genetics—where entity status requires confirmation beyond a single family—we classify *PIK3R5*‐related ataxia as a candidate association pending independent replication rather than a formal subtype. The original observation is not in question, only its nosological rank.

## The Oculomotor Phenotype: Beyond “Oculomotor Apraxia”

The term “oculomotor apraxia” (OMA) was coined by Cogan in 1952 for children with impaired initiation of voluntary horizontal saccades, compensated by characteristic jerky head thrusts toward the target.^21^ In the eye‐movement literature the term has a settled and useful meaning: a specific difficulty in launching voluntary saccades, frequently—though not invariably—accompanied by head movements in the same direction, sometimes amounting to head thrusts that bring the eyes onto target.^22^ So defined, OMA is encountered well beyond the AOA spectrum, in Joubert syndrome and other pediatric‐onset disorders and in acquired conditions such as Huntington's disease, corticobasal syndrome and bilateral hemispheric lesions.^13^, ^22^, ^23^ It is, in short, a sign shared by many disorders rather than a discrete nosological entity,^24^ yet it remains embedded in the disease name itself—which is what warrants scrutiny.

At the bedside, OMA is sought by having the patient shift gaze rapidly between two targets, looking for delayed saccade initiation, hypometric “staircase” saccades, and compensatory head movements.^24^ Saccade initiation, however, is only one part of a much wider oculomotor picture. In AT, clinical and oculographic studies have documented gaze‐evoked, downbeat and periodic alternating nystagmus, square‐wave jerks and microsaccadic oscillations, alongside impaired smooth pursuit, gaze holding, convergence, and vestibulo‐ocular reflex and its cancellation, with both reflexive and voluntary saccades hypometric and of prolonged latency.^9^, ^15^, ^25^, ^26^, ^27^ In ATLD, video‐oculography in two siblings showed hypermetric and abnormally fast saccades, saccadic intrusions and macrosaccadic oscillations, and spontaneous jerk, gaze‐evoked and rebound nystagmus.^28^ Head thrusts, meanwhile, are neither universal nor the most sensitive marker: patients with AOA2 may show markedly abnormal saccades and impaired eye–head coordination without overt thrusts.^10^ Mariani et al found that virtually all genetically confirmed patients have some quantitative oculomotor abnormality, even where the classic apraxic pattern is not evident clinically.^8^ The oculomotor profile is therefore a continuum of saccade‐initiation dysfunction, from subtle delay to frank failure, set within a broader disturbance of ocular motor control—not a binary sign (Table S2).

Because the oculomotor phenotype is broad, variable, and not specific to these disorders, naming the whole group after one sign gives that sign a prominence the evidence does not support; gene‐based nomenclature avoids this while leaving the bedside term untouched. The diagnostic value of the sign is unchanged: by whatever name, its recognition should prompt AFP and albumin measurement and genetic testing.

## A Variable Sign: Should it Define the Disease?

Terminology aside, there is a separate and more practical question: how often is the sign actually there? In many patients it is not. In the largest AOA1 series, OMA was present in only 61% of cases—prompting the explicit caveat that “OMA may be lacking, despite the misleading name of AOA1.”^6^ In AOA2 its prevalence is 34–51%,^29^ and at least one genetically confirmed ATLD patient had none.^14^ Even where video‐oculography detects abnormality in nearly everyone, only about one‐third of genetically confirmed patients show the substantially prolonged saccade latencies that constitute the clinically recognizable apraxic pattern.^8^ Where OMA does occur in AOA1 it tracks with disease severity and mutation type (83% with truncating versus 17% with missense variants), marking neurodegenerative burden rather than a defining molecular mechanism.^6^ Reported prevalences and the accompanying oculomotor findings are collated in Table S2.^33^, ^34^

Taken together, OMA is neither constant nor defining: it is absent in a third to two‐thirds of patients depending on subtype, varies with disease stage and genotype, and—when present—lies on a continuum rather than at a discrete threshold. It is therefore better regarded as a supportive than an obligatory diagnostic criterion. Its detection remains genuinely useful, prompting AFP and albumin testing and genetic confirmation; but the work‐up of progressive cerebellar ataxia with sensorimotor neuropathy should proceed whether or not an oculomotor disturbance can be demonstrated. Treating the sign as obligate risks missing the diagnosis in the substantial subset of patients in whom it never appears.

## Conclusions

Three conclusions follow. First, AOA is better understood as a single, mechanistically coherent group—the DNA damage response ataxias—ordered by prevalence and named by gene, incorporating AT and ATLD, with *PIK3R5*‐related ataxia (AOA3) held as a candidate pending replication. Second, the oculomotor phenotype of these disorders is broader than the single sign their name highlights, encompassing nystagmus, saccadic intrusions, and abnormalities of pursuit and the vestibulo‐ocular reflex. Third, the sign is inconsistent—absent in up to two‐thirds of patients—and should be supportive rather than obligatory, with cerebellar ataxia and sensorimotor neuropathy alone justifying biomarker and genetic testing.

## Disclosures

**Ethical Compliance Statement:** This Viewpoint is a conceptual and terminological analysis of previously published, peer‐reviewed literature; it involved no new studies of human or animal subjects and no individual patient data. Approval by an institutional review board or ethics committee was therefore not required for this work, and the analysis was conducted in accordance with the ethical principles of the Declaration of Helsinki and the recommendations of the International Committee of Medical Journal Editors. Informed patient consent was not necessary for this work. We confirm that we have read the Journal's position on issues involved in ethical publication and affirm that this work is consistent with those guidelines.

**Financial Disclosures and Conflicts of Interest:** Author disclosures are available in the Supporting Information. Additional supporting information may be found in the online version of this article at the publisher's website.

## Author Roles

(1) Research project: A. Conceptualization, B. Project administration, C. Supervision, D. Methodology; (2) Manuscript: A. Writing of the original draft, B. Writing – review & editing.

L.E.N.: 1B, 1D, 2A, 2B.

F.M.R.F.: 1A.

A.M.G.: 2A.

V.R.P.: 2A.

O.G.P.B.: 1C, 2B.

J.L.P.: 1A, 1C, 2B.

## Supporting information


**TABLE S1:** Clinical features of the AOA spectrum, ordered by relative prevalence, with reported oculomotor apraxia prevalence, biomarkers, and associated findings
**TABLE S2:** Reported oculomotor findings across the AOA spectrum

**Data S1:** Supporting Information

## Data Availability

Data sharing not applicable to this article as no datasets were generated or analysed during the current study.
