# Supplementary material for: Deconstructing Ataxia with Oculomotor Apraxia: Toward a Gene‐Based Classification and a Reappraisal of the Oculomotor Phenotype
Source: Mov Disord Clin Pract. 2026 Oct 2:10.1002/mdc3.70846. Online ahead of print. doi: 10.1002/mdc3.70846 (PMC13633394; doi:10.1002/mdc3.70846)
Supplement: Supplementary file 2 [file MDC3-9999-0-s001.docx]

**Supplementary Materials**

***Table S1. Clinical Features of the AOA Spectrum Ordered by Relative Prevalence***

| **Type** | **Gene** | **Age of Onset** | **OMA Prevalence** | **Key Biomarkers** | **Neuropathy** | **Other Features** | **References** |
| --- | --- | --- | --- | --- | --- | --- | --- |
| **AT** | *ATM* (11q22–23) | 2–3 years | ~30% (19/63; apraxia of horizontal gaze) | ↑ AFP >65 µg/L; radiosensitivity; ↓ lymphocytes | Sensorimotor | Telangiectasias; immunodeficiency; cancer predisposition; dystonia (89%), choreoathetosis (89%), myoclonus (92%), tremor (74%), parkinsonism (41%) | Levy & Lang 2018 (Mov Disord) [12]; Mariani et al. 2017 (Sci Rep) [8]; Farr et al. 2002 (Am J Ophthalmol) [20] |
| **AOA2** | *SETX* (9q34) | ~14 years (10–22) | 34–51% | ↑ AFP 15–65 µg/L (>90%); normal albumin; ↑ CPK (variable) | Axonal sensorimotor (97.5%) | Most common AOA in Europe/Brazil; chorea or myoclonus may be initial feature; pyramidal signs, skeletal deformities, pes cavus | Anheim et al. 2009 (Brain) [3]; Yang et al. 2025 (J Neurol) [19] |
| **AOA4** | *PNKP* (19q13) | ~4 years | Present in most | Variable: AFP mildly ↑; albumin normal/↓; cholesterol normal/↑ | Axonal sensorimotor | Prevalent in Portugal/Brazil; early dystonia (may resolve); cognitive impairment, obesity; may mimic hereditary chorea | Bras et al. 2015 (Am J Hum Genet) [21]; da Costa et al. 2022 (Mov Disord) [1] |
| **AOA1** | *APTX* (9p13) | ~7 years (4–18) | ~61% | ↓ Albumin (62%); ↑ cholesterol (75%); ↑ AFP <15 µg/L (41%) | Severe axonal sensorimotor (93%) | Chorea at onset (may resolve); prevalent in Portugal/Japan; wheelchair dependence 10–15 yrs after onset | Le Ber et al. 2003 (Brain) [22]; Renaud et al. 2018 (JAMA Neurol) [6] |
| **ATLD1** | *MRE11A* (11q21) | Early childhood (later than AT) | 61–83% | Normal AFP; radiosensitivity | Sensorimotor axonal | No telangiectasias or immunodeficiency; slower progression than AT; hypergonadotropic hypogonadism; marked phenotypic variability | Bajek et al. 2023 (Front Neurol) [13]; Yoshida et al. 2014 (Am J Med Genet) [23] |
| **ATLD2** | *PCNA* (20p12) | Early childhood | Mild (reported case) | Normal AFP | Variable | Photosensitivity, short stature, neurodegeneration; ± hearing loss; <5 cases reported | Magrino et al. 2023 (J Biol Chem) [24]; Raslan et al. 2021 (Mov Disord Clin Pract) [15] |
| **AOA5*** | *XRCC1* (19q13) | Infancy–adulthood | Variable | AFP/albumin/cholesterol: normal or not reported | Axonal sensorimotor (variable) | *Ultra-rare; few reported cases; oculomotor findings range from OMA to subtle pursuit defects | Hoch et al. 2017 (Nature) [4]; O’Connor et al. 2018 (JNNP) [14] |
| **AOA3†** | *PIK3R5* (17p12–13) | 12–18 years | ≥3/4 affected | ↑ AFP 32–99 µg/L; normal cholesterol | Axonal sensory | †Single consanguineous family; phosphoinositide signaling (not DNA repair); candidate association pending replication | Al Tassan et al. 2012 (Hum Mutat) [7] |

*OMA: oculomotor apraxia; AFP: alpha-fetoprotein; CPK: creatine phosphokinase.*

***Table S2. Reported Oculomotor Findings Across the AOA Spectrum***

| **Entity (gene)** | **Oculomotor apraxia prevalence** | **Other reported oculomotor findings** | **Principal source(s)** |
| --- | --- | --- | --- |
| **AT** (*ATM*) | ~30% (apraxia of horizontal gaze; 19/63) | Gaze-evoked, downbeat and periodic alternating nystagmus; square-wave jerks; microsaccadic oscillations; impaired pursuit, gaze holding, convergence, VOR and VOR cancellation; hypometric saccades of prolonged latency | Farr et al. 2002 [20]; Lewis et al. 1999 [27]; Shaikh et al. 2009 [28]; Brueggemann et al. 2022 [29]; Tang & Shaikh 2019 [30]; Stell et al. 1989 [31] |
| **AOA1** (*APTX*) | ~61% (83% truncating vs 17% missense variants) | Prolonged saccade latency and hypometria on video-oculography | Renaud et al. 2018 [6]; Mariani et al. 2017 [8] |
| **AOA2** (*SETX*) | 34–51% | Abnormal saccade metrics and eye–head coordination, which may occur without overt head thrusts | Anheim et al. 2009 [3]; Yang et al. 2025 [19]; Panouillères et al. 2013 [9] |
| **AOA4** (*PNKP*) | Present in most reported patients | Limited quantitative oculomotor data | Bras et al. 2015 [21]; da Costa et al. 2022 [1] |
| **ATLD1** (*MRE11A*) | 61–83% (case without OMA reported) | Hypermetric and abnormally fast saccades; saccadic intrusions and macrosaccadic oscillations; spontaneous jerk, gaze-evoked and rebound nystagmus | Bajek et al. 2023 [13]; Yoshida et al. 2014 [23]; Federighi et al. 2017 [32] |
| **ATLD2** (*PCNA*) | Mild OMA in the reported case | Limited quantitative oculomotor data | Raslan et al. 2021 [15] |
| **AOA5** (*XRCC1*) | Variable (classic OMA to subtle pursuit defects) | Findings range from classic OMA to subtle pursuit defects | Hoch et al. 2017 [4]; O’Connor et al. 2018 [14] |
| **AOA3** (*PIK3R5*) | ≥3/4 affected family members | Limited quantitative oculomotor data | Al Tassan et al. 2012 [7] |
| **Pooled VOG cohort** (AOA1/AOA2/AT) | Full apraxic pattern (markedly prolonged saccade latencies) in ~1/3 | Some quantitative oculomotor abnormality in nearly all genetically confirmed patients | Mariani et al. 2017 [8] |

*OMA: oculomotor apraxia; VOG: video-oculography; VOR: vestibulo-ocular reflex. Figures reflect the principal cohort or review for each entity and are not strictly comparable, owing to differences in ascertainment, patient age, and method of oculomotor assessment. The pooled video-oculography estimate refers to the subset showing the full apraxic pattern with markedly prolonged saccade latencies.*
